# Species- and strain-level diversity of Corynebacteria isolated from human facial skin

**DOI:** 10.1186/s12866-023-03129-9

**Published:** 2023-11-28

**Authors:** Mie Gammelgaard Jensen, Lejla Svraka, Elena Baez, Michael Lund, Anja Poehlein, Holger Brüggemann

**Affiliations:** 1 Department of Biomedicine, Faculty of Health, Aarhus University, Aarhus, Denmark; 2 Department of Genomic and Applied Microbiology, Institute of Microbiology and Genetics, University of Göttingen, Göttingen, Germany

**Keywords:** *Corynebacterium*, Skin microbiome, Antimicrobial resistance, Pan-genome, *Cutibacterium acnes*

## Abstract

**Background:**

Sequencing of the human skin microbiome revealed that *Corynebacterium* is an ubiquitous and abundant bacterial genus on human skin. Shotgun sequencing further highlighted the microbial “dark matter” of the skin microbiome, consisting of microorganisms, including corynebacterial species that were not cultivated and genome-sequenced so far. In this pilot project, facial human skin swabs of 13 persons were cultivated to selectively obtain corynebacteria. 54 isolates were collected and 15 of these were genome-sequenced and the pan-genome was determined. The strains were biochemically characterized and antibiotic susceptibility testing (AST) was performed.

**Results:**

Among the 15 sequenced strains, nine different corynebacterial species were found, including two so far undescribed species, tentatively named “*Corynebacterium vikingii*” and “*Corynebacterium borealis*”, for which closed genome sequences were obtained. Strain variability beyond the species level was determined in biochemical tests, such as the variable presence of urease activity and the capacity to ferment different sugars. The ability to grow under anaerobic conditions on solid agar was found to be species-specific. AST revealed resistances to clindamycin in seven strains. A *Corynebacterium pseudokroppenstedtii* strain showed additional resistance towards beta-lactam and fluoroquinolone antibiotics; a chromosomally located 17 kb gene cluster with five antibiotic resistance genes was found in the closed genome of this strain.

**Conclusions:**

Taken together, this pilot study identified an astonishing diversity of cutaneous corynebacterial species in a relatively small cohort and determined species- and strain-specific individualities regarding biochemical and resistance profiles. This further emphasizes the need for cultivation-based studies to be able to study these microorganisms in more detail, in particular regarding their host-interacting and, potentially, -beneficial and/or -detrimental properties.

**Supplementary Information:**

The online version contains supplementary material available at 10.1186/s12866-023-03129-9.

## Background

Knowledge about the skin microbiome and its individual microbial members is crucial to understand their beneficial contributions to skin health. The microbiome of oily, moist and dry areas of the skin is often dominated by the genera *Cutibacterium* and *Staphylococcus*, as well as other genera such as *Corynebacterium* [1–3]. This genus comprises more than 130 species [4, 5]. A few *Corynebacterium* species are considered pathogens, including *Corynebacterium diphtheriae*, the primary cause of diphtheria, *Corynebacterium ulcerans* and *Corynebacterium pseudotuberculosis*. Regarding skin commensals, many different corynebacterial species have been identified so far, such as *Corynebacterium simulans, Corynebacterium striatum, Corynebacterium accolens, Corynebacterium tuberculostearicum, Corynebacterium fastidiosum, Corynebacterium afermentans, Corynebacterium kroppenstedtii, Corynebacterium pseudokroppenstedtii, Corynebacterium amycolatum, Corynebacterium resistens, Corynebacterium aurimucosum*, and *Corynebacterium jeikeium* [4, 5]. Different anatomical locations vary regarding corynebacterial abundances. Areas with higher humidity such as the axillary vault, toe web, popliteal fossa, and groin are thought to be preferred environments for growth, but other sites, such as dry and oily sites are also colonized with corynebacteria [1–3]. Currently, there is limited knowledge regarding cutaneous corynebacteria, in part due to their fastidious and slow growth, which makes cultivation more challenging, compared to other bacteria e.g., staphylococci [6, 7].

Cutaneous corynebacteria seem to have diverse host protective functions. Colonization resistance can be achieved by some corynebacterial species/strains, mediated by antimicrobial compounds such as bacteriocins found in e.g., *C. jeikeium* [8] and predicted to be produced by many other coynebacteria [9]. Other mechanisms of interference were found in *C. striatum*, which is able to reduce the virulence of *Staphylococcus aureus* by inhibiting the accessory gene regulator (*agr*) quorum sensing system [10], and in *C. accolens*, whose lipase activity leads to free fatty acid production that can inhibit *Streptococcus pneumoniae* [11, 12].

Despite being one of the most abundant genera of the skin microbiome, knowledge regarding cutaneous corynebacteria down to species level, as well as species and strain properties, remain scarce. Moreover, recent studies suggested the presence of additional, so far uncharacterized corynebacterial species on human skin [13]. Thus, this pilot study aimed at gathering knowledge on cutaneous corynebacteria by cultivation from facial skin swabs and subsequent genome sequencing of isolates. In addition, biochemical assays and drug susceptibility testing were carried out as well as interaction studies with *Cutibacterium acnes*.

## Methods

### Cohort and sample acquisition

Swab samples were collected from 13 volunteers (female, *n* = 11; male, *n* = 2) with an age range of 21–58 years from forehead and cheek skin. None of the participants had been on antibiotic treatment for the past 6 months before sample collection. An area of 25 cm^2^ of forehead and cheek skin was sampled with a cotton swab, which was pre-moistened in aqueous sampling buffer (50 mM TrisHCL and 1 mM EDTA). The volunteers were recruited in Aarhus, Denmark.

### Cultivation

The samples were inoculated on agar media and incubated in an aerobic atmosphere containing 5% CO_2_. The agar media was Furazolidone, Tween-80, Oil red O agar (FTO) medium [14] with the following composition: 40 g trypticase soy agar (TSA), 5 g yeast extract, 10 ml Tween-80, 1 L ultra-filtrated-water; after autoclaving furazolidone (6 µg/ml) and 1 ml of Oil red O (0.5% stock solution) was added. Bacterial growth on FTO agar was examined for up to four days. Orange-pink circular colonies were suspected to be corynebacteria [14]. Two to four bacterial colonies of the described morphology were selected from each plate and stored as stocks (Brain heart infusion (BHI) medium with 10% glycerol stored at -80 °C).

### Genomic DNA extraction

The MasterPure™ Gram Positive DNA Purification Kit (Lucigen) was used according to the manufacturer’s instructions. DNA quality and yield was checked by agarose gel electrophoresis along with concentration determination using the Qubit® dsDNA HS Assay Kit (Life Technologies GmbH, Darmstadt, Germany).

### 16S rRNA gene fragment amplification and sequencing

The V1-V3 region of the 16S rRNA gene was amplified with the following primers: 5’-TATTACCGCGGCTGCTGGCA-3’ and 5’-TCAGATTGAACGCTGGCGGC-3’. A PCR reaction mixture of 25 µl containing 8 µl sterile PCR grade water, 2 µl primer mix (5 µmol each), 5 µl of the 1:100 diluted DNA, and 10 µl of 5Prime Hotmaster mix (Quanta Bio) was prepared. The following PCR scheme was used: 94 °C for 5 min (1 cycle), 94 °C for 1 min, 60 °C for 1 min (30 cycles), 72 °C for 2 min, and 72 °C for 8 min (1 cycle). The PCR products were verified on agarose gels. Sequencing of the PCR products using the V1-V3 primers was done at Eurofins Genomics (Ebersberg, Germany). Sequence comparison with the NR database (status: May 2023) at NCBI was done using blastn. A sequence identity > 99% of the amplicon sequence with a database entry led to species assignment.

### Genome sequencing

Illumina shotgun libraries were prepared using the Nextera XT DNA Sample Preparation Kit and subsequently sequenced on a MiSeq system using the v3 reagent kit with 600 cycles (Illumina, San Diego, CA, United States) as recommended by the manufacturer. Quality filtering was done with version 0.39 of Trimmomatic [15]. Assembly was performed with version 3.15.2 of the SPAdes genome assembler software [16]. Version 2.2.1 of Qualimap was used to validate the assembly and determine the sequence coverage [17]. Default parameters were used for all mentioned software unless otherwise specified. In total, 15 corynebacterial strains were sequenced with a genome coverage of 51- to 237-fold (in average 160-fold).

The genome sequences of four strains (P3-F1, P4-C1, P8-C1, P15-C1) were closed. For Nanopore sequencing, 1.5 µg unsheared HWD was used for the library preparation using the ligation sequencing kit 1D (SQK-LSK109) and the native barcode expansion kit (EXP-NBD103). Sequencing was performed for 72 h on a MinION Mk1B device with a SpotON R9.4.1 flow cell, using MinKNOW v19.06.8 and Guppy v3.2.1 for base calling (Oxford Nanopore, Oxford, UK). Unicycler v0.4.6 [18] was used to perform the hybrid assembly, resulting in one circular replicon per strain. The closed circular chromosomes have sizes of 2,118,088 bp (P3-F1), 2,313,418 bp (P4-C1), 2,355,242 bp (P8-C1) and 2,512,499 bp (P15-C1). All draft and closed genome sequences were deposited in GenBank. The accession numbers can be found here: PRJNA991496 (12 draft genomes); CP129965 (closed genome of “*C. vikingii*” P3-F1); CP129966 (closed genome of “*C. borealis*” P4-C1); CP129967 (closed genome of “*C. borealis*” P8-C1); CP137757 (closed genome of *C. pseudokroppenstedtii* P15-C1).

### Bioinformatics tools

Gene prediction and annotation of all genomes were performed with RAST [19]. The eggnog-Mapper was used for additional annotation [20]. For phylogenomic analyses, the core genome was identified and aligned with the Parsnp program from the Harvest software package [21]. Corynebacterial genomes available from GenBank (status May 2023) were used along with the 15 corynebacterial genomes from this study to build a core genome-based phylogeny. Reliable core genome single-nucleotide variants identified by Parsnp were used for the reconstruction of genome-based phylogeny using FastTree 2 [22]. Phylogenetic trees were visualized (as unrooted trees) using the Interactive Tree Of Life [23]. BRIG was used for genome comparison and visualization [24]. For the BRIG analysis the following additional genomes were used (GenBank accession numbers in brackets): *C. appendicis* DSM44531 (GCA_900156665), *C. genitalium* ATCC33030 (GCA_000143825), *C. aurimucosum* ATCC700975 (GCA_000022905), *C. striatum* 215 (GCA_002803965). The Comprehensive Antibiotic Resistance Database (CARD) was used to analyze genomes for antimicrobial resistance determinants [25]. JSpeciesWS was used for average nucleotide identify (ANI) calculations [26]. AntiSMASH (version 7.0) was used to predict gene clusters for secondary metabolites [27].

For the pan-genome analysis, proteinortho [28] was used as a tool to detect orthologous genes in the genomes of 28 different corynebacterial strains. Besides the 15 strains isolated here, type strains of the here identified nine species and close relatives were used (GenBank accession number in brackets): *C. appendicis* CIP107643 (GCA_030408415); *C. appendicis* DSM44531 (GCA_900156665); *C. aurimucosum* ATCC700975 (GCA_000022905); *C. bovis* 4826 (GCA_003932295); *C. genitalium* ATCC33030 (GCA_000143825); *C. kefirresidentii* FDAARGOS1055 (GCA_016599755); *C. kroppenstedtii* DSM44385 (GCA_000023145); *C. pseudokroppenstedtii* UMB3152 (GCA_030217185); *C. sanguinis* CCUG58655 (GCA_007641235); *C. striatum* 215 (GCA_002803965); *C. striatum* FDAARGOS1115 (GCA_016728105); *C. tuberculostearicum* FDAARGOS1117 (GCA_016728365); *C. ureicelerivorans* IMMIBRIV2301 (GCA_000747315). The applied bidirectional blastp thresholds were: protein identity ≥ 25%; protein coverage ≥ 50%; e-value ≤ 1e-05 (loose threshold); and protein identity ≥ 50%; protein coverage ≥ 75%; e-value ≤ 1e-05 (strict threshold).

### Biochemical tests

Enzymatic activities and fermentation abilities of corynebacterial strains were tested with the API® CORYNE system (Biomerieux). The system comprised 19 tests, including 11 tests for enzymatic activities. These activities included nitrate reduction, pyrazinamidase, pyrrolidonyl-arylamidase, alkaline phosphatase, β-glucosidase, β-glucuronidase, β-galactosidase, α-glucosidase, N-acetyl-β-glucosaminidase, urease and hydrolysis of gelatin. Fermentation of eight sugars, namely glucose, ribose, xylose, mannitol, maltose, lactose, sucrose and glycogen were tested. The tests were carried out following the instructions of the manufacturer. Bacterial suspensions with a turbidity greater than 6 McFarland were prepared, which were then used to inoculate the 11 enzymatic tests. For the fermentation tests, 0.5 mL of the suspension was transferred to the API® GP medium, and the suspension was added to the last nine wells on the strip. The fermentation wells and the urea well were sealed with mineral oil. The strips were incubated under aerobic conditions for 24 h at 37 °C. Subsequently, specific reagents were added to the wells according to the manufacturer instructions and results were recorded after 10 min. The API® CORYNE test was done in triplicates.

### Antibiotic susceptibility testing

Antibiotic susceptibility testing was done to evaluate the susceptibility of the 15 corynebacterial strains to a panel of commonly used antibiotics, namely penicillin, ciprofloxacin, clindamycin, vancomycin, rifampin, and doxycycline. The disc diffusion assay (DDA) was applied according to EUCAST instructions (https://www.eucast.org/). In brief, bacterial suspensions with a turbidity of 0.5 McFarland were prepared. DDA was performed on FTO agar plates that lacked furazolidone. After inoculation of the plates and a 15 min drying time, the antibiotic discs were gently pressed onto the plates using a sterile tweezer. The plates were then incubated for 24 h under aerobic conditions. The DDA was done in duplicates.

### Antagonistic plate assay and anaerobic growth test

The following *C. acnes* strains were used as lawn bacteria in antagonistic plate assays: 12.1.L1 (SLST type A1, an acne isolate) and P31 (SLST type F4, an isolate from a prosthetic joint infection) [29, 30]. Reinforced Clostridial Agar (RCA) was used for agar-based cultivation of *C. acnes* strains, in an anaerobic atmosphere at 37 °C for 3 days. To prepare *C. acnes* lawn bacteria, cultures of *C. acnes* strains were made in BHI broth supplemented with 1% Tween-80 (BHIT medium), cultivated for 48 h under anaerobic conditions. For the inoculum, the cultures were diluted with pre-warmed BHIT medium to an OD_600nm_ of 0.1. One mL of the bacterial suspension was distributed onto FTO agar (without furazolidone and Oil-Red O), which were air-dried before the addition of corynebacterial stab cultures. The corynebacterial strains were cultivated on FTO agar, and liquid grown was done in BHIT medium for 24 h aerobically at 37 °C. The bacterial culture was diluted to an OD_600nm_ of 0.1. A volume of 5 µl of each corynebacterial suspension was added to the lawn plate. Incubation of the plates was performed anaerobically and recorded for up to 4 days. The antagonistic assays were done in triplicates. In order to test if the 15 corynebacterial strains were able to grow under anaerobic conditions, a volume of 5 µl of each corynebacterial suspension (OD_600nm_ of 0.1) was added to FTO plates without furazolidone and Oil red O and incubated anaerobically for 4 days.

## Results

### Isolation and identification of cutaneous corynebacteria

The forehead and cheek skin of 13 healthy participants aged 21–58 years (average age 32.5 years) were swabbed. All 26 skin swabs were cultivated on FTO plates for four days. Two to four colonies (orange-pink color) were selected from each plate (Fig. S1). All isolated colonies were subjected to 16S rRNA gene fragment (V1-V3 region) amplification and sequencing. Subsequent sequence comparison using BLAST revealed that in 24 out of 26 skin swab samples corynebacteria could be isolated, in total 54 corynebacterial isolates (Table S1). Based on the BLAST results, the most often detected species were *C. tuberculostearicum* in seven participants, *C. sanguinis* in four participants and *C. kroppenstedtii* in three participants. In six participants, corynebacterial isolates were found that could not be unambiguously assigned to a known corynebacterial species (Table S1).

### Genomic diversity among isolated cutaneous corynebacteria

To get more insights in cutaneous corynebacteria, 15 isolates were selected for whole genome sequencing. The selection was based on 16S rRNA species assignment with the aim to cover a range of different corynebacterial species. The isolates originated from nine different participants (Table S1). The GC content of the corynebacterial genomes ranged from 56.1 to 72.8%, the genome size range was 2037 kb to 2597 kb and the number of protein-coding sequences (CDS) ranged from 1987 to 2453 (Table 1).

**Table 1:** Genome sequence statistics of 15 cutaneous corynebacterial isolates

Strain	Species V1-V3 result	Species ANI result	GC (%)	contigs	Size (bp)	CDS**
P1-C1	*C. sp.*	C. kroppenstedtii *	56.1	6	2,456,372	2218
P1-F1	*C. sanguinis*	*C. sanguinis*	65.4	44	2,339,157	2231
P3-F1	*C. tuscaniense*	novel species *(”C. vikingii”)*	63.1	1	2,118,088	1987
P4-C1	*C. appendicis*	novel species *(”C. borealis”)*	64.1	1	2,313,418	2179
P4-C2	*C. ureicelerivorans*	*C. ureicelerivorans*	65.6	16	2,037,780	1998
P4-F2	*C. appendicis*	novel species *(”C. borealis”)*	64.1	46	2,313,042	2202
P5-C4	*C. tuberculostearicum*	*C. kefirresidentii*	58.3	35	2,509,220	2453
P5-F2	*C. tuberculostearicum*	C. tuberculostearicum *	59.9	30	2,303,461	2181
P7-C1	*C. sanguinis*	*C. kefirresidentii*	58.5	104	2,402,275	2395
P7-F1	*C. tuberculostearicum*	*C. kefirresidentii*	58.5	14	2,409,602	2317
P8-C1	*C. appendicis*	novel species *(”C. borealis”)*	64.0	1	2,355,242	2221
P12-C2	*C. bovis*	*C. bovis*	72.8	382	2,597,258	2398
P14-F4	*C. tuberculostearicum*	*C. ureicelerivorans*	65.5	29	2,114,939	2058
P15-C1	*C. kroppenstedtii*	*C. pseudokroppenstedtii*	57.2	1	2,512,499	2286
P15-C2	*C. sanguinis*	*C. sanguinis*	65.3	65	2,329,888	2265

* these isolates had an average nucleotide identity (ANI) below 95% to the *C. kroppenstedtii* and *C. tuberculostearicum* reference genomes (Table S2)

** RAST annotation

**Fig. 1:**
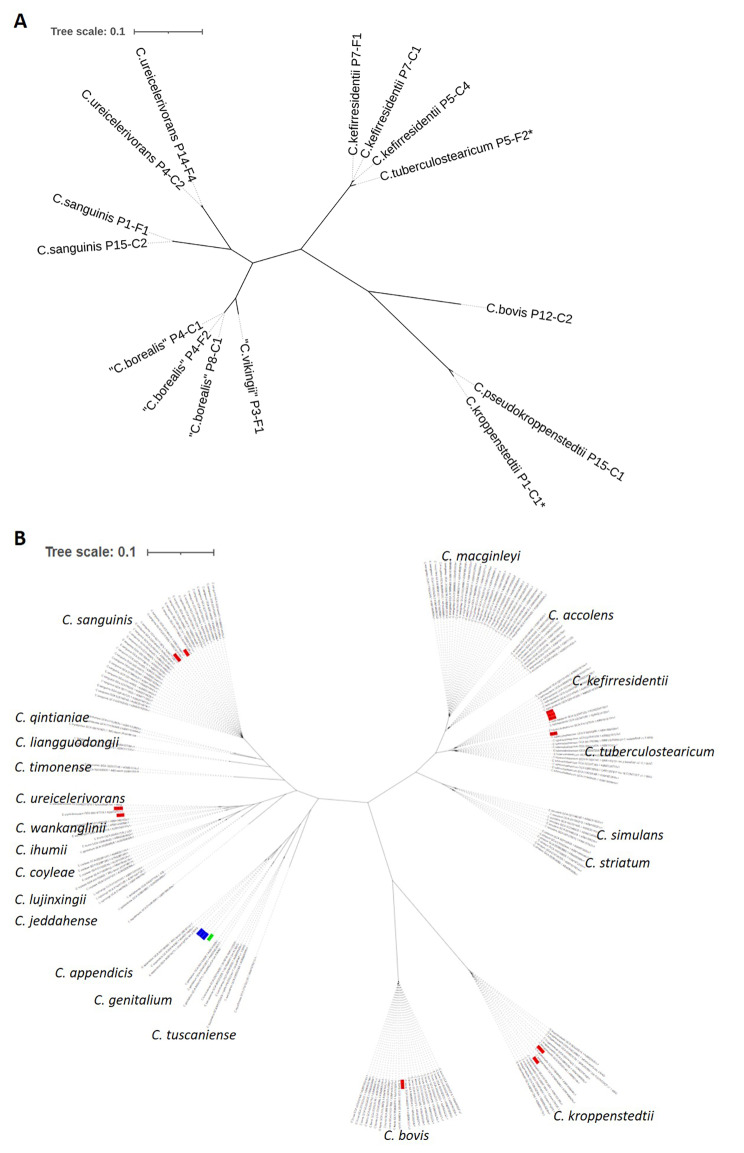
Core genome-based phylogeny of cutaneous corynebacteria sequenced in this study. **(A)** 15 strains isolated from normal skin were compared. Core genome alignment was done with Parsnp and a phylogenetic tree was built with FastTree 2. **(B)** The 15 genomes (three strains of the novel species “*C. borealis”* in blue; one strain of “*C. vikingii”* in green; the other 11 strains in red) were compared to other corynebacterial genomes taken from NCBI.

A phylogenetic tree based on core genome comparison was built, showing the diversification among the 15 isolates into four larger clades (Fig. 1A). In addition, the 15 genomes sequenced here were phylogenetically compared to other corynebacterial genomes (the closest available relatives) taken from public databases (Fig. 1B). Eleven isolates could be assigned to known corynebacterial species. Four isolates were distantly related to *C. appendicis, C. genitalium* and *C. tuscaniense.*

**Fig. 2:**
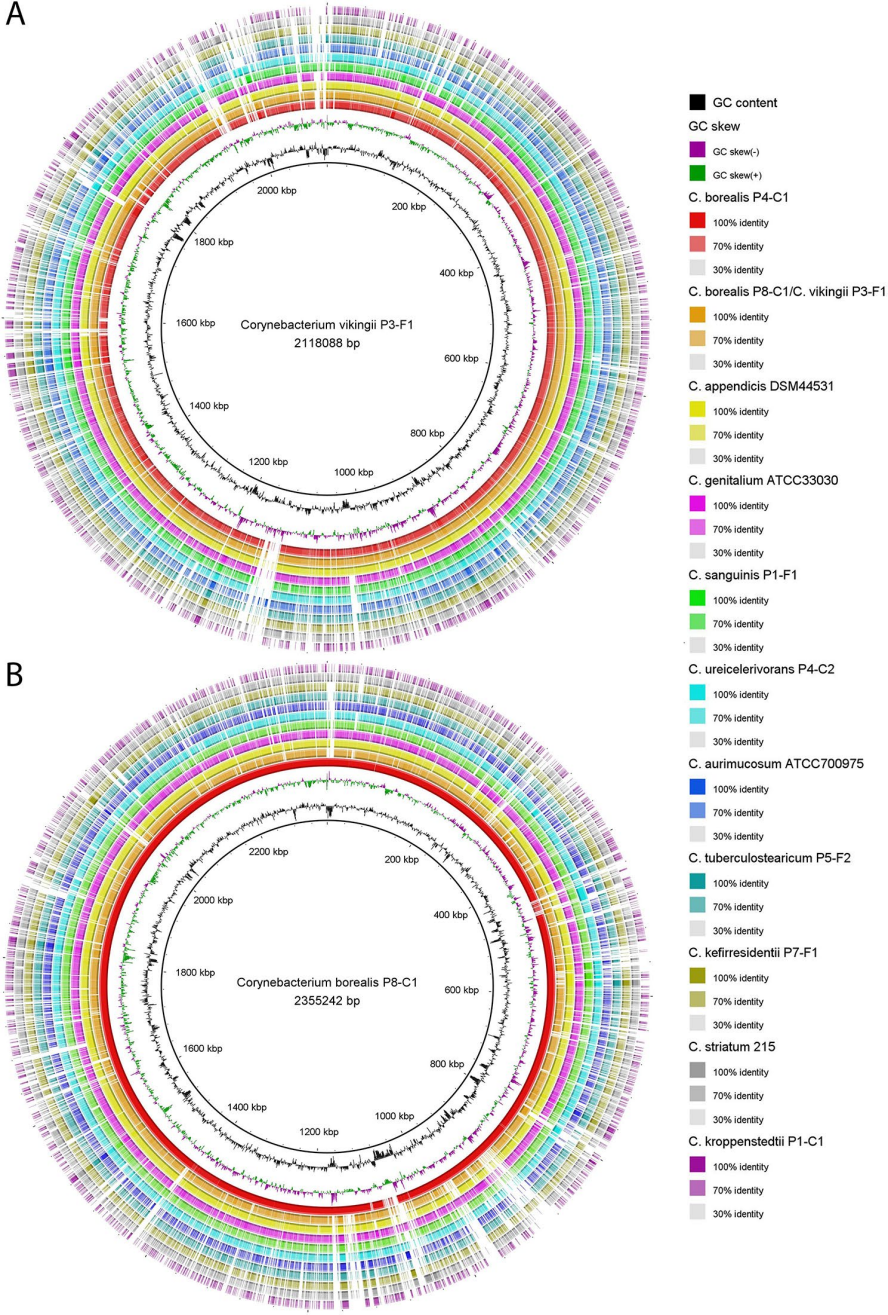
Closed genomes of two novel species of cutaneous corynebacteria, tentatively named “*C. vikingii”* and “*C. borealis****”***. **(A)** Comparative genome maps of “*C. vikingii”* (strain P3-F1) and **(B)** “*C. borealis”* (strain P8-C1). Reference genomes of closely related species, i.e., *C. appendicis* and *C. genitalium* were used, as well as more distant genomes, i.e., from the species *C. sanguinis, C. ureicelerivorans, C. aurimucosum, C. tuberculostearicum, C. kefirresidentii, C. striatum* and *C. kroppenstedtii*. Accession numbers of the genomes are given in the methods part. Each colored ring represents a genome that was compared to “*C. vikingii”* P3-F1 (in A) and “*C. borealis”* P8-C1 (in B). Blast nucleotide identify thresholds were set to 100%, 70% and 30%, and color-coded as indicated in the legend. The same legend applies to both A and B, with the exception that the orange ring represents the genome of “*C. borealis*” P8-C1 in A and “*C. vikingii*” P3-F1 in B

A closer look, using genome-wide average nucleotide identities (ANI), identified nine different corynebacterial species among the 15 isolates (Table S2A), applying a 95% ANI cutoff for species separation [31]. Correct species assignment was done by whole genome comparison and ANI calculation with respective reference genomes, if available (Table S2B). This showed that only nine isolates could be unambiguously (ANI > 95%) assigned to a known species i.e., the four species *C. bovis* (1 isolate), *C. kefirresidentii* (3 isolates), *C. ureicelerivorans* (2 isolates), *C. sanguinis* (2 isolates) and *C. pseudokroppenstedtii* (1 isolate). Another two isolates had *C. tuberculostearicum* (P5-F2) and *C. kroppenstedtii* (P1-C1) as close relatives with ANIs of 94.5% and 89.2%, respectively (Table S2B). The remaining four isolates with ANIs < 85% to any other so far sequenced corynebacterial species could be regarded as novel species. The closest relative of these four isolates was *C. appendicis* with ANIs of 82.2–84.1% (Table S2B). The four isolates could be further separated into two new species, hereafter tentatively named “*Corynebacterium vikingii*” (isolate P3-F1) and “*Corynebacterium borealis*” (isolates P4-C1, P4-F2, and P8-C1). “*C. vikingii”* and “*C. borealis”* showed an ANI of 82.3% to each other, indicating a close evolutionary background (Table S2A, Fig. 1). Genomes of one isolate of “*C. vikingii”* and two isolates of “*C. borealis”* were closed with Nanopore sequencing. Comparison of the closed genomes with other corynebacterial genomes showed a large overall similarity and synteny between cutaneous corynebacteria, with species- and strain-specific gene clusters (Fig. 2).

### Pan-genome analysis of cutaneous corynebacterial strains

**Fig. 3:**
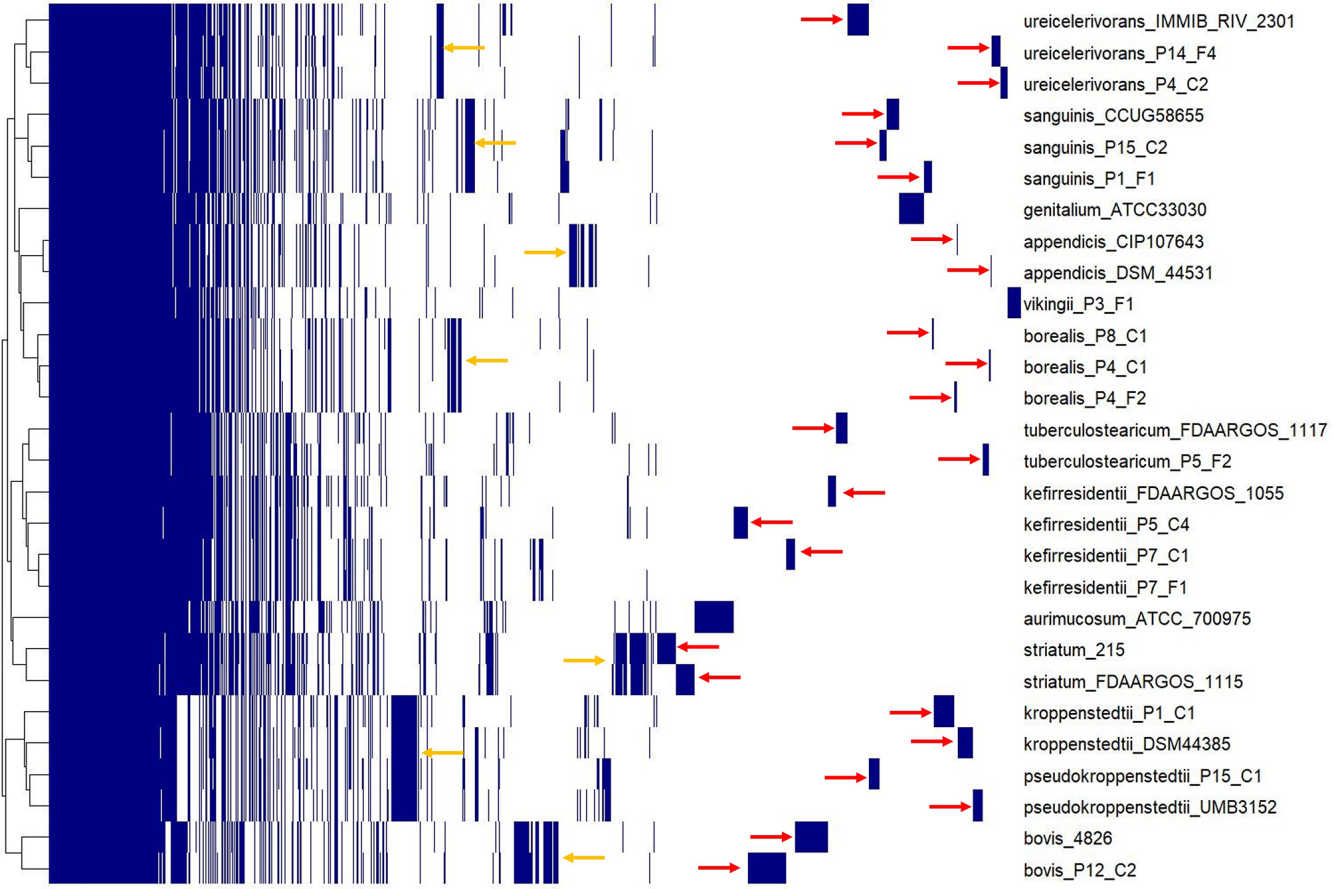
Pan-genome analysis of 28 corynebacterial strains belonging to 13 different species. Shown is the analysis with the loose blastp threshold (≥ 25% protein identity and ≥ 50% sequence coverage), with a pan-genome comprised of 8708 CDS. Species- and strain-specific gene clusters were identified (orange and red arrows, respectively; only shown for species, which are represented with more than one strain). 983 CDS (11.2% of all pan-genome CDS) constitute the core genome (present in all 28 strains). 3257 CDS (37.4%) are strain-specific (present only in one strain)

To further get insights into species- and strain-specific functions a pan-genome analysis was carried out using a bidirectional blastp approach with the program proteinortho [28]. Genomes of 28 strains belonging to 13 different species were used, including the 15 here sequenced strains, and, in addition, 13 reference genomes of the same or closely related species. A loose (≥ 25% protein identity and ≥ 50% sequence coverage) and a strict (≥ 50% protein identity and ≥ 75% sequence coverage) threshold were used (Table S3). The analyses resulted in a pangenome of 8708 (loose) and 12,289 (strict) CDS, respectively (Fig. 3, Table S3). The core genome (CDS present in all 28 genomes) comprises 983 and 639 CDS, applying the loose and strict thresholds, respectively. More than 1/3 of the CDS of the pangenome were strain-specific (in both, loose and strict analyses). This reflects the diversity of the species and strains.

We had a closer look at the two new species “*C. borealis*”and “*C. vikingii*”: 187 and 175 CDS were “*C. borealis*”- and “*C. vikingii*”-specific (strict threshold), respectively (Table S4). Regarding predicted functions, 136 of the 187 “*C. borealis*”-specific proteins and 132 of the 175 “*C. vikingii*”-specific proteins were annotated as hypothetical proteins, underlining the lack of knowledge regarding this species and, likely, cutaneous corynebacteria in general. Regarding proteins with predicted functions, “*C. borealis”-*specific functions included restriction-modification systems, phage-related proteins, toxin-antitoxin systems, transposases, (stress-responsive) transcriptional regulators, sortase, glycosyltransferase, and multiple transport functions (MFS type; ABC-type; siderophore-related; predicted substrates: chromate; oligopeptide, alanine, nitrate sulfonate bicarbonate, potassium). Similar functional categories were found for “*C. vikingii*”-specific CDS. In addition, there is a non-ribosomal peptide synthetase (NRPS) cluster specific for “*C. vikingii*” (Fig. S2).

### Differences among cutaneous corynebacteria regarding enzymatic and metabolic properties

**Fig. 4:**
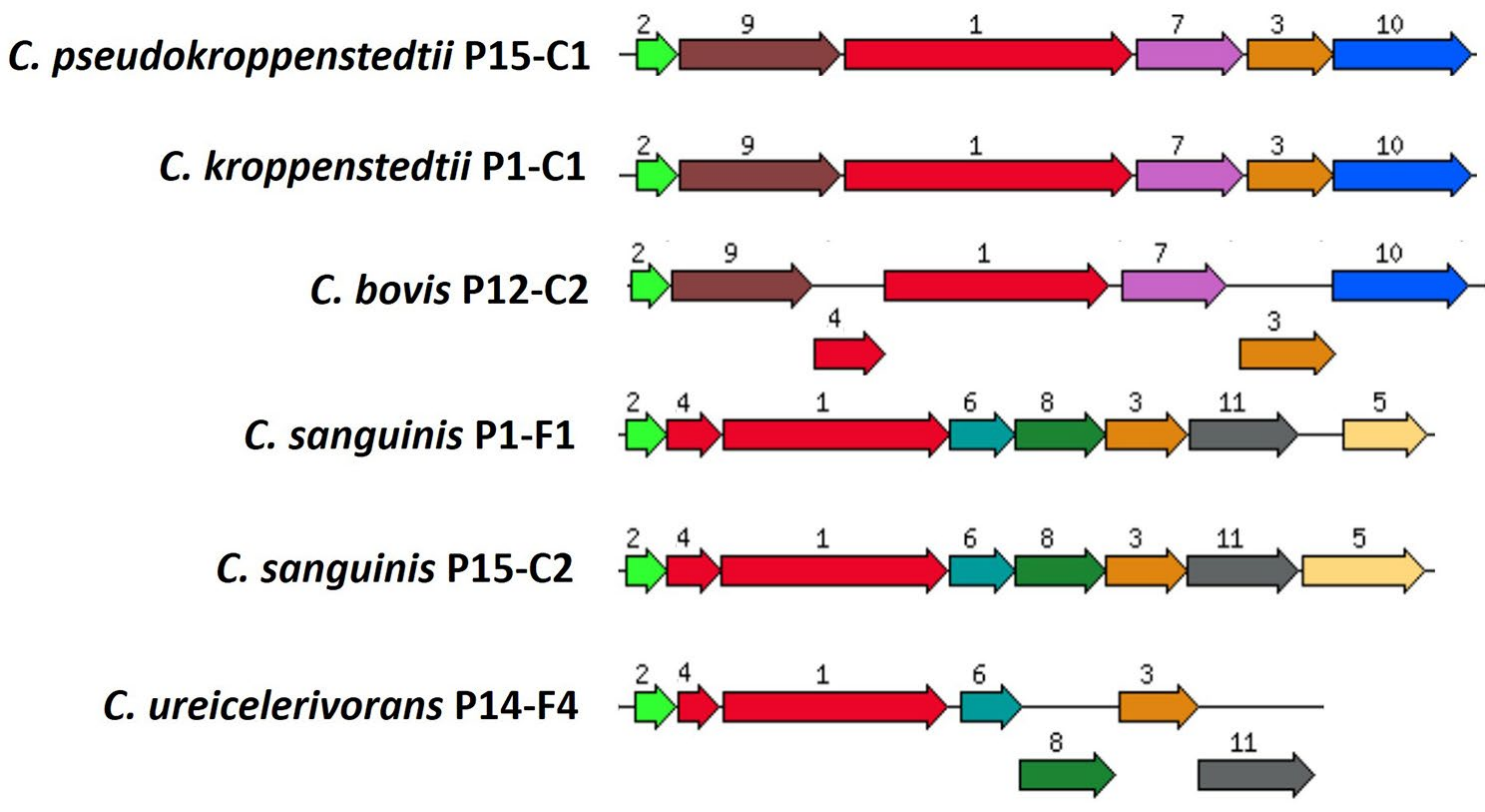
Urease gene clusters in corynebacteria sequenced in this study. The following urease genes were found in the six corynebacterial strains with urease activity: 1, *ureC* (urease alpha subunit); 2, *ureA* (urease gamma subunit); 3, *ureG* (urease accessory protein); 4, *ureB* (urease beta subunit); 6, *ureE* (urease accessory protein); 7 and 8, *ureF* (urease accessory protein); 10 and 11, *ureD* (urease accessory protein). Other urease gene cluster-associated genes: 5, gene encoding urea transporter; 9, gene encoding conserved hypothetical protein. The genes encoding the alpha and beta urease subunits (*ureC* and *ureB*) are fused in *C. kroppenstedtii* P1-C1 and *C. pseudokroppenstedtii* P15-C1. The gene *ureE* is absent in *C. kroppenstedtii* P1-C1, *C. pseudokroppenstedtii* P15-C1 and *C. bovis* P12-C2.

Next, we wanted to know if the cutaneous corynebacterial isolates had differing biochemical properties. Therefore, biochemical tests were applied (API™ Coryne) to test for enzymatic activities and sugar-degrading capacities (Table 2). All strains were negative for the following reactions: reduction of nitrate, β-glucuronidase, β-galactosidase, α-glucosidase, N-acetyl-β-glucosaminidase, and fermentation of xylose, mannitol, lactose, glycogen. Most strains were positive for pyrazinamindase (except *C. bovis* P12-C2 and “*C. vikingii”* P3-F1), alkaline phosphatase (except “*C. vikingii”* P3-F1 and *C. pseudokroppenstedtii* P15-C1), hydrolysis of gelatin (except *C. sanguinis* P1-F1) and fermentation of glucose (except *C. ureicelerivorans* P4-C2, *C. bovis* P12-C2, and two of the three isolates of “*C. borealis”*). Regarding other reactions, there is a high degree of strain-specific variability. For example, the urease reaction is positive in six out of 15 strains. To get a first insight into the genomic basis for urease activity differences among the tested strains, we searched for urease genes in the 15 genomes. Urease-encoding genes were previously identified in *Corynebacterium glutamicum* [32]. All six urease-positive strains carried the genes for the urease (subunits *ureABC*) and urease accessory polypeptides (*ureDEFG*), and all nine urease-negative strains lacked the respective genes (Fig. 4). Two different urease gene cluster structures were found: the two *C. sanguinis* strains and *C. ureicelerivorans* P14-F4 carried the genes *ureABC* and *ureEFGD*, whereas *C. kroppenstedtii* P1-C1, *C. pseudokroppenstedtii* P15-C1 and *C. bovis* P12-C2 harbored *ureAXBC* and *ureFGD* (Fig. 4). As another example, saccharose fermentation was only carried out by six strains; all these six strains carried a gene encoding sucrose-6-phosphate hydrolase (*sacC*).

**Table 2:** Biochemical properties of the 15 cutaneous corynebacterial isolates

strain	species	PYZ	PyrA	PAL	ESC	URE	GEL	GLU	RIB	MAL	SAC
P1-F1	*C. sanguinis*	+	-	+	-	+	-	+	-	-	-
P15-C2		+	-	+	-	+	+	+	+	-	-
P4-C2	*C. ureicelerivorans*	+	+	+	-	-	+	-	-	-	-
P14-F4		+	+	+	-	+	+	+	-	-	-
P5-C4	*C. kefirresidentii*	+	+	+	-	-	+	+	+	-	-
P7-F1		+	+	+	-	-	+	+	+	-	+
P7-C1		+	+	+	-	-	+	+	+	-	+
P12-C2	*C. bovis*	-	+	+	-	+	+	-	-	-	-
P5-F2	*C. tuberculostearicum*	+	+	+	-	-	+	+	+	-	+
P1-C1	*C. kroppenstedtii*	+	-	+	-	+	+	+	-	-	-
P15-C1	*C. pseudokroppenstedtii*	+	-	-	+	+	+	+	-	-	-
P3-F1	“*C. vikingii”*	-	+	-	-	-	+	+	-	-	-
P4-C1	“*C. borealis”*	+	-	+	-	-	+	-	-	+	+
P8-C1		+	-	+	-	-	+	-	-	-	+
P4-F2		+	-	+	-	-	+	+	+	-	+

Abbreviations: PYZ (pyrazinamindase); PyrA (pyrrolidonyl acrylamidase); PAL (alkaline phosphatase); ESC (β–glucosidase, esculin); URE (urease); GEL (hydrolysis of gelatin); fermentation of glucose (GLU), ribose (RIB), maltose (MAL), and saccharose (SAC)

### Resistance to clindamycin is common in cutaneous corynebacteria

Antibiotic susceptibility testing (AST) using the disc diffusion test was performed for all 15 sequenced corynebacterial isolates. The following antibiotics were tested: penicillin, clindamycin, ciprofloxacin, vancomycin, rifampicin, doxycycline (Table 3). All strains were sensitive to vancomycin, rifampicin and doxycycline. One isolate (P15-C1, *C. pseudokroppenstedtii*) was resistant to three antibiotics: clindamycin, penicillin and ciprofloxacin (Fig. S3). Besides isolate P15-C1, six additional strains were resistant to clindamycin (Table 3).

Looking at the genomic basis of the identified resistances, four strains (“*C. vikingii”* P3-F1, “*C. borealis*” P4-F2, *C. ureicelerivorans* P14-F4, *C. pseudokroppenstedtii* P15-C1) carried the *ermX* gene, encoding a 23S rRNA methyltransferase (Table S5). The two clindamycin-resistant *C. sanguinis* strains P1-F1 and P15-C2 carried an alternative 23S rRNA methyltransferase similar to *erm*38/39/40 (Table S5). The genome of the multi-resistant *C. pseudokroppenstedtii* strain P15-C1 was closed to get solid information regarding the genomic basis of its multi-resistant phenotype. The genome carried a 17 kb gene cluster on the chromosome that harbored *ermX*, three genes for aminoglycoside phosphotransferases (*aph*(3’), *aph*(3’’), *aph*(6)) and a gene encoding an MFS efflux pump, predicted to be responsible for chloramphenicol resistance (Table S5).

**Table 3:** Antibiotic susceptibility testing of 15 corynebacterial isolates against six antibiotics

species	strain	Penicillin	Ciprofloxacin	Clindamycin	Vancomycin	Rifampin	Doxycycline
*C. sanguinis*	P1-F1	-	-	+	-	-	-
	P15-C2	-	-	+	-	-	-
*C. ureicelerivorans*	P4-C2	-	-	-	-	-	-
	P14-F4	-	-	+	-	-	-
*C. kefirresidentii*	P5-C4	-	-	-	-	-	-
	P7-F1	-	-	-	-	-	-
	P7-C1	-	-	-	-	-	-
*C. bovis*	P12-C2	-	-	+	-	-	-
*C. tuberculostearicum*	P5-F2	-	-	-	-	-	-
*C. kroppenstedtii*	P1-C1	-	-	-	-	-	-
*C. pseudokroppenstedtii*	P15-C1	+	+	+	-	-	-
“*C. vikingii”*	P3-F1	-	-	+	-	-	-
“*C. borealis”*	P4-C1	-	-	-	-	-	-
	P8-C1	-	-	-	-	-	-
	P4-F2	-	-	+	-	-	-

EUCAST breakpoints (resistance): penicillin < 29 mm; ciprofloxacin < 25 mm; vancomycin < 17 mm; clindamycin < 20 mm; rifampicin < 30 mm; doxycycline (tetracycline) < 24 mm

### Anaerobic growth and interference of corynebacteria with *Cutibacterium acnes*

**Fig. 5:**
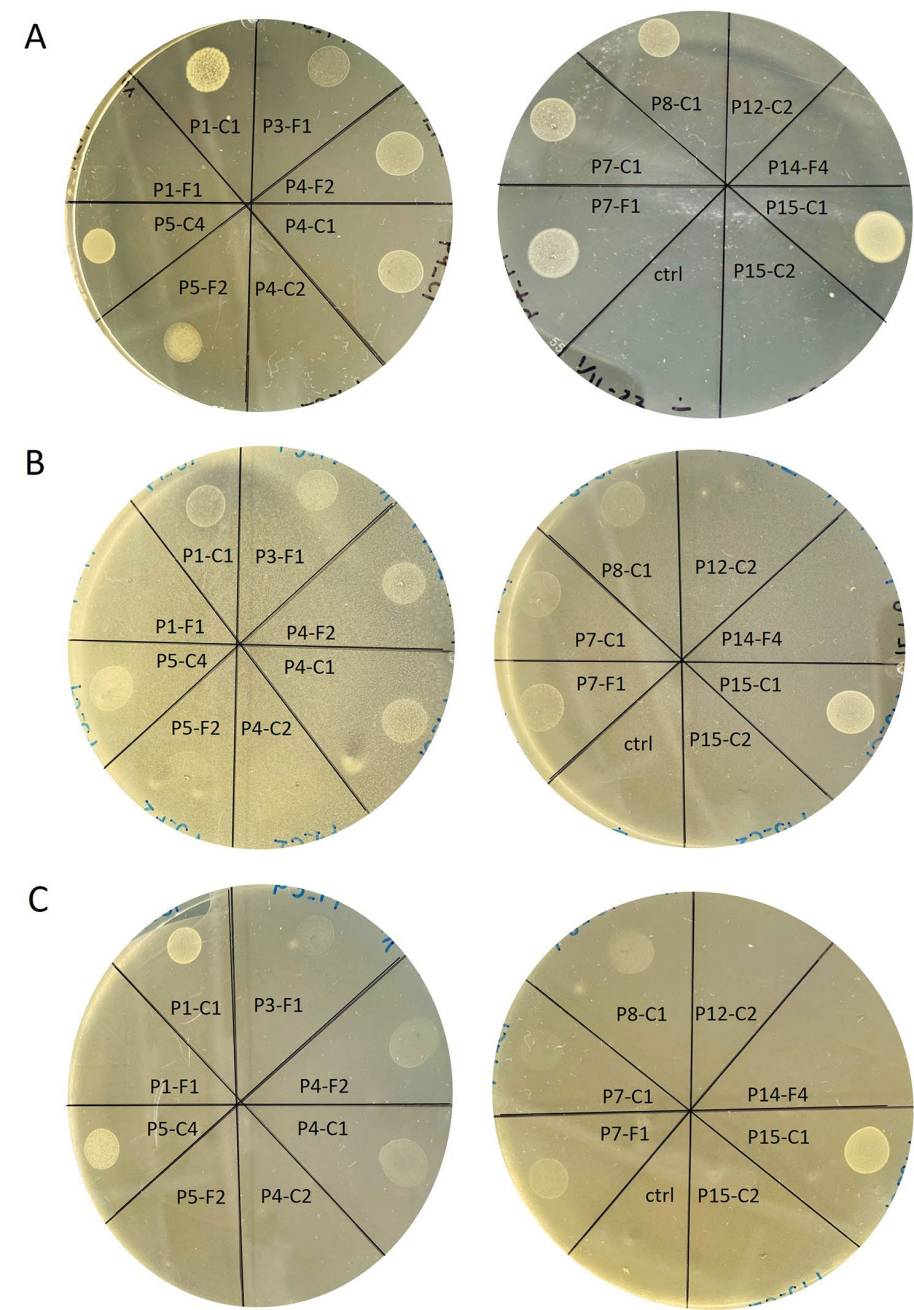
Evaluation of anaerobic growth of 15 corynebacterial strains on solid media and antagonistic plate assay with *C. acnes*. **A.** 15 genome-sequenced corynebacterial strains were tested for their anaerobic growth ability. Five strains could not grow under anaerobic conditions on the used agar plates. **B**. *C. acnes* strain 12.1.L1 (SLST type A1) as lawn and the corynebacterial strains as stab culture. **C**. *C. acnes* strain P31 (SLST type F4) as lawn. Corynebacterial species names: see Table 2; ctrl, medium control

We determined potential interferences between the 15 sequenced corynebacterial strains and the prominent and abundant skin colonizer *Cutibacterium acnes*. An antagonistic assay on solid media was performed under anaerobic conditions. Two disease-associated *C. acnes* strains, 12.1.L1 (SLST type A1) and P31 (SLST type F4) [29, 30], were used as lawn on agar plates, while the 15 *Corynebacterium *sp. strains were added as stab cultures. In addition, anaerobic growth tests of the 15 corynebacterial strains were performed: not all corynebacterial strains were able to grow under anaerobic conditions on solid media (Fig. 5A). The two *C. sanguinis* strains (P1-F1, P15-C2), the two *C. ureicerlerivorans* strains (P4-C2, P14-F4) and *C. bovis* P12-C2 were unable to grow. In contrast, the strains of the species *C. kroppenstedtii* (P1-C1), *C. pseudokroppenstedtii* (P15-C1), *C. kefirresidentii* (P5-C4, P7-F1, P7-C1) and “*C. borealis*” (P4-C1, P4-F2, P8-C1) exhibited good growth under these conditions. The antagonistic plate assay showed that the two *C. acnes* strains were unable to inhibit the stab culture growth of most corynebacterial strains (Fig. 5B and 5C). One exception was C. *tuberculostearicum* P5-F2 that was inhibited by both *C. acnes* strains.

## Discussion

This pilot study investigated the diversity of human skin-associated corynebacteria by selective cultivation from facial skin swabs and subsequent molecular and biochemical analyses of the obtained isolates. All 13 participants carried facial corynebacteria and in the majority of forehead and cheek samples isolates belonging to more than one corynebacterial species were found.

Species assignment of the obtained 54 isolates by partial 16S rRNA gene sequencing (V1-V3 region) was in several cases not sufficient to identify the corynebacterial species correctly. This was in part due to high sequence similarity of the targeted 16S rRNA gene region between different corynebacterial species, but also due to the lack of reference sequences for some isolates/species that have not previously been cultivated, identified and/or described. This problem has been reported in previous (skin) microbiome sequencing projects, sometimes referred to as microbial “dark matter”, i.e., the presence of sequences that cannot be (correctly) assigned (e.g., to species level) due to the absence of microbial reference sequences/genomes [2, 13, 33, 34]. Our study identified four isolates, belonging to two so far uncharacterized corynebacterial species that have not been isolated/described before, to our knowledge. We have tentatively named these species “*C. vikingii*” and “*C. borealis*”. A recent study has also cultivated and genome-sequenced 41 corynebacterial isolates from skin sites [13]. We have compared these 41 genomes to the 15 genomes sequenced here and could find some overlap, in particular regarding isolates belonging to the species *C. kefirresidenti* and C. *tuberculostearicum* (Fig. S4). However, isolates belonging to the here identified two novel species “*C. vikingii*” and “*C. borealis*” were not found in the study of Saheb Kashaf et al. (Fig. S4B); instead, different, potentially novel species were identified in their study [13]. Overall, this highlights the lack of knowledge about human-associated corynebacteria and further underlines the high genomic diversity, which is in stark contrast to the genus *Cutibacterium*, for which only five cutaneous species are known, with *C. acnes* as by far the most dominating one [2, 3, 35].

The genomic diversity is also a reason for differences in biochemical reactions of cutaneous corynebacteria as found in this study. The biochemical diversity is apparent among different corynebacterial species but also among strains belonging to the same species. For example, hydrolysis of gelatin and ribose fermentation differed in the two strains of *C. sanguinis*, urease activity and fermentation of glucose differed in the two strains of *C. ureicelerivorans*, and the three strains of “*C. borealis*” differed regarding the fermentation of glucose, ribose and maltose. These results suggest that biochemical tests are only partially useful for assigning isolates to corynebacterial species.

Besides biochemical differences among the isolates, we also noticed some variation regarding susceptibility to the lincosamide antibiotic clindamycin. Resistance to clindamycin was reported in other corynebacteria [36, 37] and was linked to the presence of *ermX*, encoding a 23S rRNA methyltransferase that also confers resistance to macrolides such as erythromycin in other bacteria, e.g. *C. acnes* [38]. Seven out of 15 tested corynebacterial strains were resistant to clindamycin. Four of them, all belonging to different species (“*C. vikingii”*, “*C. borealis*”, *C. ureicelerivorans, C. pseudokroppenstedtii*) carried the *ermX* gene. The other strains of “*C. borealis*” and *C. ureicelerivorans* were sensitive to clindamycin and lacked *ermX*, again highlighting strain variability within corynebacterial species. The *C. pseudokroppenstedtii* strain P15-C1 was resistant to multiple antibiotics, including penicillin, clindamycin and ciprofloxacin. Similar multi-resistance phenotypes were previously predicted in specific strains of *C. accolens* and *C. striatum* [39–41]. The resistance genes (*erm*X, *aph*(3’), *aph*(3’’), *aph*(6) and a gene encoding an MFS efflux pump, predicted to be responsible for chloramphenicol resistance) were clustered in a 17 kb region that seems to be a region of high genome plasticity, since it harbors multiple repeats (five 841 bp repeats) and nine genes encoding mobile elements proteins or derivatives thereof (IS6, IS3 and Tn3 family transposases). This region has a high sequence similarity with parts of the 28.3 kb resistance plasmid pJA144188 of *Corynebacterium resistens* DSM 45100 [42], including the macrolide-lincosamide-streptogramin resistance region and the chloramphenicol and aminoglycoside resistance region; the latter region has a high similarity to the Tn*45* family transposon Tn*5717a* from the pathogen *Corynebacterium urealyticum* DSM 7109.

We also tested for possible interference between corynebacteria and *C. acnes* strains. Previous studies have highlighted bacterial interferences on the skin, such as *C. acnes* versus staphylococci [43, 44]. In addition, a strong negative correlation between *Cutibacterium* and *Corynebacterium* on human skin was noted [45]. The mechanisms of such possible interferences are poorly understood. Strains of *C. acnes* belonging to the SLST class H and L have been shown to produce a bacteriocin, termed cutimycin that has antimicrobial activity [43]. In addition, metabolic end products of *C. acnes* such as short chain fatty acids (propionate, acetate, butyrate and valerate) might be able to inhibit certain acid-sensitive bacterial strains, similar to previous findings reporting the inhibitory effect of propionic acid on *Staphylococcus aureus* [46]. There is very little knowledge regarding possible antimicrobial activities of corynebacteria. Previously identified antimicrobial compounds produced by cornynebacteria include corynicin JK of *C. jeikeium* [8], ulceracin 378 of *C. ulcerans* [47] and corynaridin of *Corynebacterium lactis* [48]; the latter was shown to be active against *C. acnes*. A genome analysis of the here isolated 15 corynebacterial strains identified several predicted gene clusters for secondary metabolite biosynthesis (Fig. S2). For instance, genes encoding non-ribosomal peptide synthetases (NRPS) could be identified in strains of “*C. borealis*”, “*C. vikingii”, C. kefirresidentii, C. bovis, C. kroppenstedtii* and *C. pseudokroppenstedtii*, but similarities to known NRPS were very low, indicating that these corynebacterial strains likely produce so far uncharacterized compounds. The suitability of experimental approaches to identify bacterial interferences very much depends on the applied media and conditions. The genera *Cutibacterium* and *Corynebacterium* have different growth preferences regarding oxygen, with corynebacteria being aerobes or facultative anaerobes and *C. acnes* being a “nanaerobe”, i.e., an organism that does not require oxygen for growth, but can benefit from the presence of nanomolar concentrations of oxygen. Thus, assays to assess interferences are difficult to set up to accommodate these differences in growth conditions. The antagonistic assay used here was done under anaerobic conditions. Ten of the 15 corynebacterial strains could grow facultative anaerobically on solid media. Only one *C. tuberculostearicum* strain was inhibited by *C. acnes* under the applied conditions and none of the corynebacterial strains were able to inhibit *C. acnes*. The applied cultivation conditions were not optimal for corynebacteria and likely favored the growth of *C. acnes.*

This study has several limitations. Only relatively few persons were sampled (n = 13); sampling was done from two facial skin sites only. The choice of these facial skin sites (rather than other, e.g., moist skin sites) related to the prospects to find novel probiotic strains that could be used in topical skin applications. Another limitation was that we randomly selected 2–4 colonies per agar plate only, thus have likely missed other corynebacterial species. In addition, we used one specific agar medium only (FTO agar). Given the here observed variability in biochemical properties among cutaneous corynebacteria, additional media and, potentially, alternative cultivation conditions might be favorable for other corynebacterial species/strains. The interference tests were only carried out on solid agar media, and anaerobic conditions were chosen.

## Conclusions

Taken together, the data highlight the ubiquity and diversity of human-associated corynebacteria. The study further emphasizes the current insufficient recovery of skin-resident corynebacteria; more (cultivation-dependent) studies are needed to obtain collections of strains (and their genomes) that represent the entire corynebacterial population of the human skin microbiota. We noticed strain-specific functionalities regarding biochemical properties, which challenges taxonomic classification based on phenotypes. Antimicrobial resistance was mainly restricted to clindamycin in the here analyzed cohort; however, resistance genes encoding rRNA methyltransferases and aminoglycoside phosphotransferases are present in a few strains that could potentially be spread by horizontal gene transfer.

### Electronic supplementary material

Below is the link to the electronic supplementary material.

Supplementary Material 1

Supplementary Material 2

Supplementary Material 3

Supplementary Material 4

Supplementary Material 5

Supplementary Material 6

Supplementary Material 7

Supplementary Material 8

Supplementary Material 9

## Data Availability

The datasets supporting the conclusions of this article are available in the GenBank repository, with the following accession numbers: PRJNA991496 (12 draft genomes); PRJNA991509 (closed genome of “*C. vikingii*” P3-F1); PRJNA991511 (closed genome of “*C. borealis*” P4-C1); PRJNA991512 (closed genome of “*C. borealis*” P8-C1).

## Abbreviations

ANI: average nucleotide identity
AST: antimicrobial susceptibility testing
FTO: Furazolidone, Tween-80, Oil red O
SLST: single-locus sequence typing
