# Supplementary figures and images for: Species- and strain-level diversity of Corynebacteria isolated from human facial skin
Source: BMC Microbiol. 2023 Nov 28;23:366. doi: 10.1186/s12866-023-03129-9 (PMC10683109; doi:10.1186/s12866-023-03129-9)

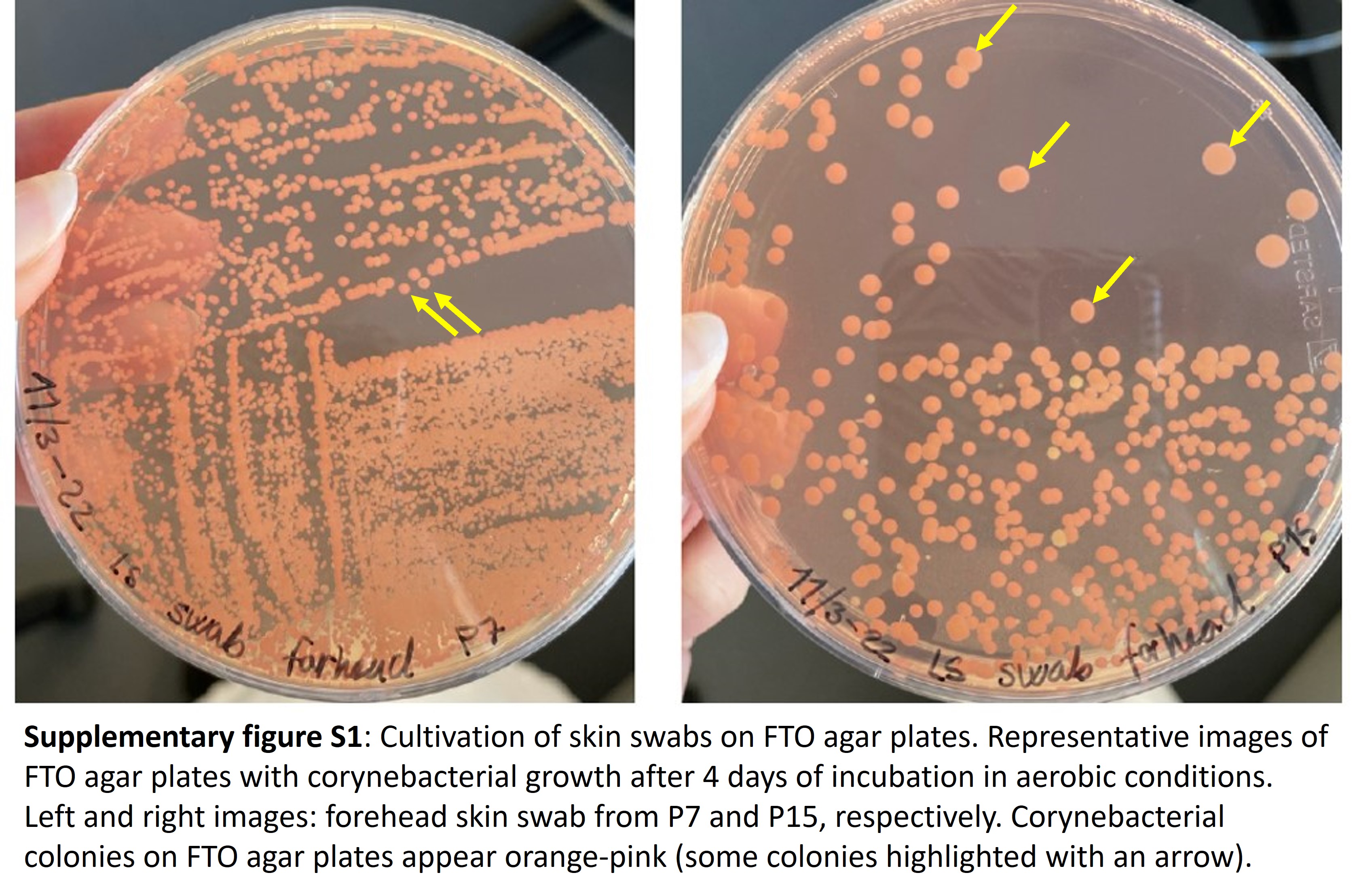

Supplement: Supplementary file 1 — Supplementary Material 1 [file 12866_2023_3129_MOESM1_ESM.jpg]

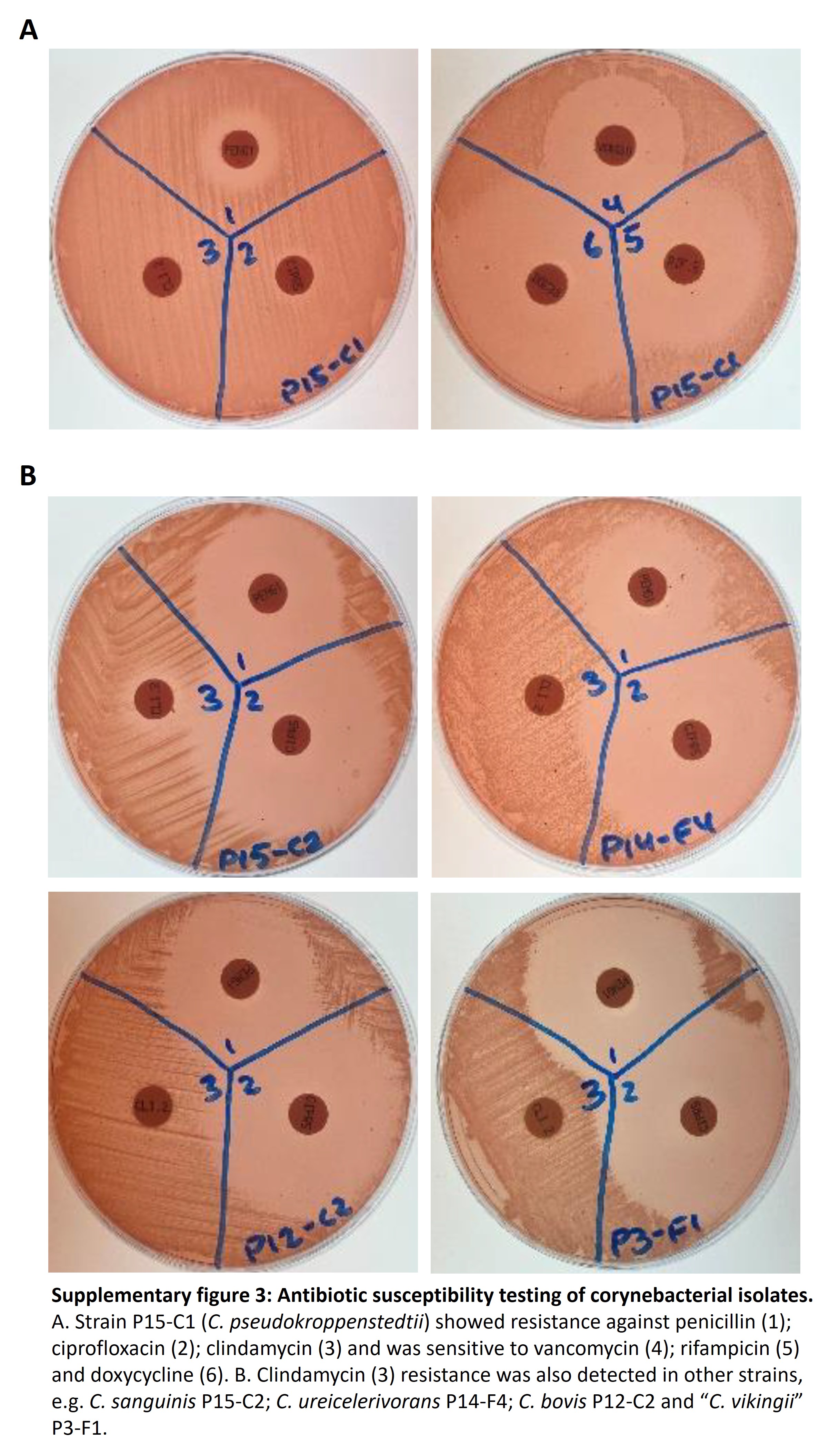

Supplement: Supplementary file 3 — Supplementary Material 3 [file 12866_2023_3129_MOESM3_ESM.jpg]

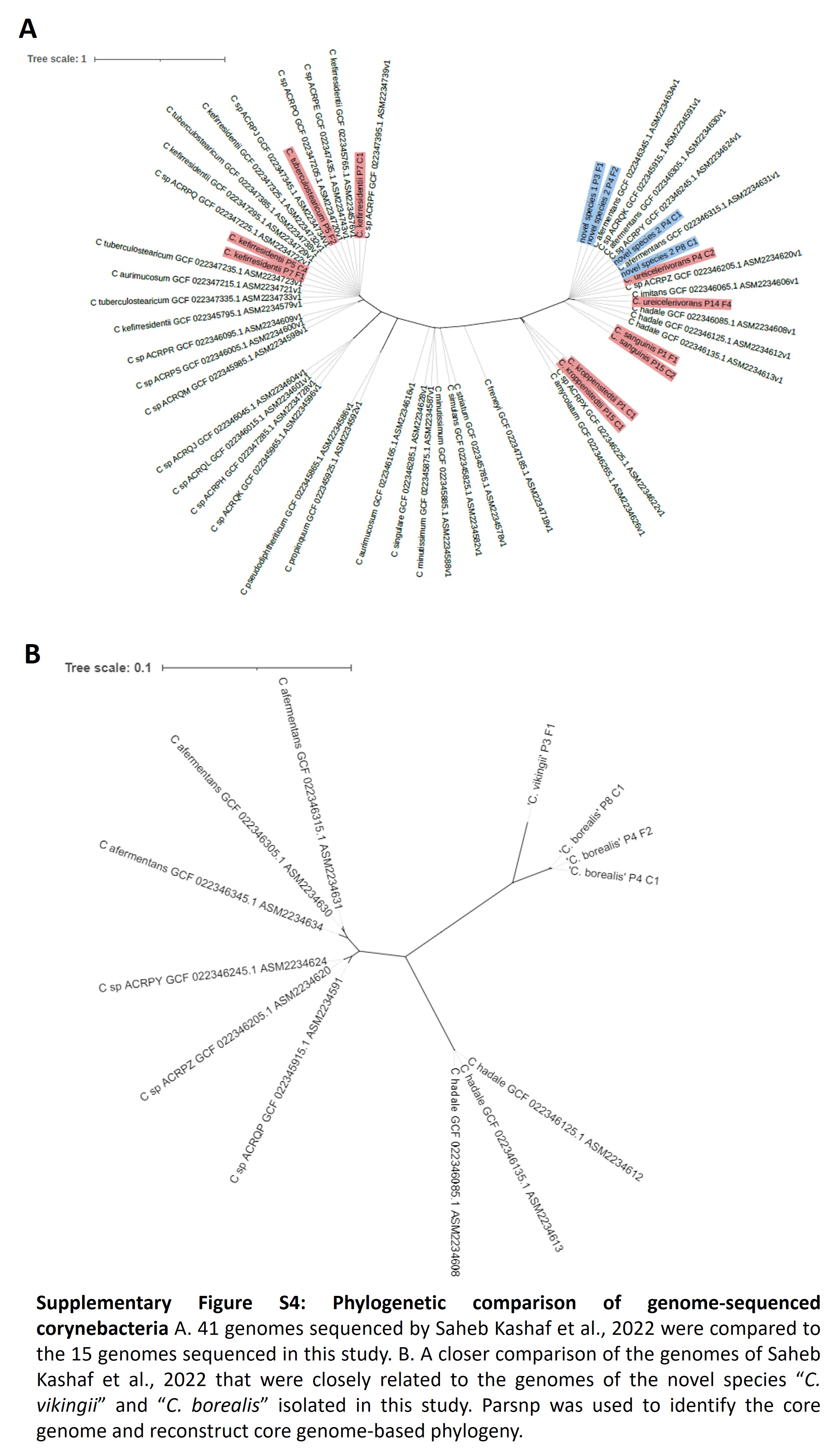

Supplement: Supplementary file 4 — Supplementary Material 4 [file 12866_2023_3129_MOESM4_ESM.jpg]
