# Supplementary material for: Species- and strain-level diversity of Corynebacteria isolated from human facial skin
Source: BMC Microbiol. 2023 Nov 28;23:366. doi: 10.1186/s12866-023-03129-9 (PMC10683109; doi:10.1186/s12866-023-03129-9)
Supplement: Supplementary file 2 — Supplementary Material 2 [file 12866_2023_3129_MOESM2_ESM.pdf]

Supplementary figure S2: Identification of secondary metabolite biosynthesis gene clusters in the 15 corynebacterial strains. AntiSMASH was used to search the genomes ('relaxed' mode).

C. borealis P4-C1

Region	Type	From	To	Most similar known cluster		Similarity
Region 1	terpene	607,454	628,323	carotenoid	Terpene	25%
Region 2	ectoine	1,068,507	1,078,881			
Region 3	NRP-metallophore, NRPS	1,322,537	1,374,912	coelichelin	NRP	45%
Region 4	T1PKS	1,834,165	1,878,952			

carotenoid biosynthetic gene cluster from *Corynebacterium glutamicum*
coelichelin biosynthetic gene cluster from *Streptomyces coelicolor* A3(2)

C. borealis P8-C1

Region	Type	From	To	Most similar known cluster		Similarity
Region 1	terpene	287,786	308,655	carotenoid	Terpene	25%
Region 2	ectoine	776,200	786,574			
Region 3	T1PKS	1,557,875	1,602,662			

carotenoid biosynthetic gene cluster from *Corynebacterium glutamicum*

C. borealis P4-F2

Region	Type	From	To	Most similar known cluster		Similarity
Region 4.1	terpene	7,584	28,453	carotenoid	Terpene	25%
Region 15.1	T1PKS	4,318	42,713			
Region 17.1	ectoine	9,366	19,740			
Region 19.1	NRP-metallophore, NRPS	1	21,194	amychelin A/amychelin B	NRP	11%

carotenoid biosynthetic gene cluster from *Corynebacterium glutamicum*
amychelin A biosynthetic gene cluster from *Amycolatopsis methanolica*

C. vikingii P3-F1

Region	Type	From	To	Most similar known cluster		Similarity
Region 1	terpene	885,451	906,323	carotenoid	Terpene	25%
Region 2	T1PKS, NRP-metallophore, NRPS	1,091,164	1,174,201	griseobactin	NRP	15%
Region 3	NI-siderophore	1,578,546	1,590,906	FW0622	Other	25%
Region 4	ectoine	1,784,981	1,795,355			

carotenoid biosynthetic gene cluster from *Corynebacterium glutamicum*
griseobactin biosynthetic gene cluster from *Streptomyces* sp. ATCC 700974
FW0622 biosynthetic gene cluster from *Verrucosispora* sp. FIM060022

C. kefirresidentii P7-F1

Region	Type	From	To	Most similar known cluster		Similarity
Region 1.1	terpene	20,827	41,921			
Region 8.1	terpene	71,542	92,417	carotenoid	Terpene	25%
Region 8.2	NAPAA	98,208	132,110	ε-Poly-L-lysine	NRP	100%
Region 9.1	T1PKS	61,592	106,295			

carotenoid biosynthetic gene cluster from *Corynebacterium glutamicum*
ε-Poly-L-lysine biosynthetic gene cluster from *Epichloe festucae*

C. kefirresidentii P7-C1

Region	Type	From	To	Most similar known cluster		Similarity
Region 8.1	terpene	23,574	44,668			
Region 13.1	T1PKS	25,162	48,897			
Region 19.1	NAPAA	1	22,889	ε-Poly-L-lysine	NRP	100%
Region 53.1	terpene	1	13,808	carotenoid	Terpene	25%

ε-Poly-L-lysine biosynthetic gene cluster from *Epichloe festucae*
carotenoid biosynthetic gene cluster from *Corynebacterium glutamicum*

C. kefirresidentii P5-C4

Region	Type	From	To	Most similar known cluster		Similarity
Region 1.1	terpene	20,691	41,785			
Region 6.1	T1PKS	52,989	97,692			
Region 8.1	terpene	72,812	93,687	carotenoid	Terpene	25%
Region 8.2	NAPAA	94,769	124,629	ε-Poly-L-lysine	NRP	100%
Region 13.1	NRPS , transAT-PKS	1	50,952			
Region 20.1	NI-siderophore	13,956	25,084	dehydroxynocardamine	NRP	28%

carotenoid biosynthetic gene cluster from *Corynebacterium glutamicum*
ε-Poly-L-lysine biosynthetic gene cluster from *Epichloe festucae*
dehydroxynocardamine biosynthetic gene cluster from *Corynebacterium propinquum*

C. kroppenstedtii P1-C1

Region	Type	From	To	Most similar known cluster		Similarity
Region 1.1	terpene	574,911	596,017			
Region 1.2	aminopolycarboxylic-acid	711,297	724,936	[S,S]-EDDS	Other	75%
Region 1.3	NRPS	1,055,948	1,097,911	diazaquinomycin H/diazaquinomycin J	Other	4%
Region 3.1	T3PKS	320,533	361,726	phenazine SA/phenazine SB/phenazine SC	Alkaloid	18%

[S,S]-EDDS(ethylenediamine-disuccinate) biosynthetic gene cluster from *Amycolatopsis japonica*
diazaquinomycin H biosynthetic gene cluster from *Micromonospora* sp. B006
phenazine SA biosynthetic gene cluster from *Streptomyces* sp.

C. pseudokroppenstedtii P15-C1

Region	Type	From	To	Most similar known cluster		Similarity
Region 1.1	terpene	566,209	587,288			
Region 2.1	T3PKS	44,088	85,281	phenazine SA/phenazine SB/phenazine SC	Alkaloid	18%
Region 2.2	NRP-metallophore , NRPS	405,760	457,938	fimsbactin A	NRP:NRP siderophore	11%

phenazine SA biosynthetic gene cluster from *Streptomyces* sp.
fimsbactin A biosynthetic gene cluster from *Acinetobacter* sp. ADP1

C. ureicelerivorans P4-C2

Region	Type	From	To	Most similar known cluster		Similarity
Region 4.1	NAPAA	85,547	119,377			
Region 6.1	terpene	52,861	73,709	carotenoid	Terpene	50%
Region 9.1	T1PKS	8,971	52,449	dehydroxynocardamine	NRP	42%

carotenoid biosynthetic gene cluster from *Corynebacterium glutamicum*
dehydroxynocardamine biosynthetic gene cluster from *Corynebacterium propinquum*

C. ureicelerivorans P14-F4

Region	Type	From	To	Most similar known cluster		Similarity
Region 3.1	terpene	167,993	188,841	carotenoid	Terpene	50%
Region 4.1	T1PKS	47,577	92,286			
Region 18.1	NAPAA	1	19,684			

carotenoid biosynthetic gene cluster from *Corynebacterium glutamicum*

C. sanguinis P15-C2

Region	Type	From	To	Most similar known cluster		Similarity
Region 3.1	NAPAA	26,768	60,547			
Region 4.1	terpene	79,004	99,837	carotenoid	Terpene	50%
Region 26.1	ectoine	16,186	26,497			
Region 32.1	T1PKS	1	23,323			

carotenoid biosynthetic gene cluster from *Corynebacterium glutamicum*

C. sanguinis P1-F1

Region	Type	From	To	Most similar known cluster		Similarity
Region 4.1	NAPAA	40,485	74,264			
Region 7.1	ectoine	16,186	26,560			
Region 10.1	terpene	72,817	88,226	carotenoid	Terpene	50%
Region 16.1	T1PKS	1	42,403			

carotenoid biosynthetic gene cluster from *Corynebacterium glutamicum*

C. tuberculostearicum P5-F2

Region	Type	From	To	Most similar known cluster		Similarity
Region 7.1	NAPAA	38,567	72,490			
Region 7.2	terpene	72,608	93,483	carotenoid	Terpene	25%
Region 10.1	T1PKS	34,603	68,232			

carotenoid biosynthetic gene cluster from *Corynebacterium glutamicum*

C. bovis P12-C2

Region	Type	From	To	Most similar known cluster		Similarity
Region 4.1	NRPS	1	31,742			
Region 33.1	terpene	1	14,705	SF2575	Polyketide:Type II polyketide+Saccharide:Hybrid/tailoring saccharide	6%
Region 40.1	T1PKS	1	15,013			
Region 43.1	NAPAA	1	14,813	ε-Poly-L-lysine	NRP	100%
Region 48.1	NRPS	1	14,202			
Region 51.1	terpene	314	13,653			
Region 64.1	NRPS	1	12,167			
Region 106.1	NRPS	1	8,504			
Region 118.1	NRPS-like	1	8,023			
Region 152.1	NRPS	1	6,289			
Region 153.1	NRPS	1	6,195			
Region 185.1	NRPS	1	4,714			
Region 211.1	NRPS	1	4,187			
Region 221.1	NRPS-like	1	3,885			

SF2575 biosynthetic gene cluster from *Streptomyces* sp. SF2575
ε-Poly-L-lysine biosynthetic gene cluster from *Epichloe festucae*

C. bovis 4826

Region	Type	From	To	Most similar known cluster		Similarity
Region 6.1	terpene	117,350	138,753	SF2575	Polyketide:Type II polyketide+Saccharide:Hybrid/tailoring saccharide	6%
Region 7.1	NRPS	30,005	83,492	amychelin A/amychelin B	NRP	11%
Region 8.1	T1PKS	35,489	80,780			
Region 8.2	NRPS	169,561	202,707	heterobactin A/heterobactin S2	NRP	18%
Region 9.1	NRPS	1	44,452			
Region 9.2	NRP-metallophore , NRPS	45,103	115,223	frankobactin A1/frankobactin A2/frankobactin A3/frankobactin B1/frankobactin B2/frankobactin B3/frankobactin C1	NRP	16%
Region 10.1	NAPAA	38,028	71,909	ε-Poly-L-lysine	NRP	100%
Region 10.2	terpene	106,381	127,187			

SF2575 biosynthetic gene cluster from *Streptomyces* sp. SF2575
amychelin A biosynthetic gene cluster from *Amycolatopsis methanolica*
heterobactin A biosynthetic gene cluster from *Rhodococcus erythropolis* PR4
frankobactin A1 biosynthetic gene cluster from *Frankia* sp. CH37
ε-Poly-L-lysine biosynthetic gene cluster from *Epichloe festucae*
